# Reducing Thermal Degradation of Perovskite Solar Cells during Vacuum Lamination by Internal Diffusion Barriers

**DOI:** 10.1021/acsaem.4c02567

**Published:** 2024-11-07

**Authors:** Robert Witteck, Duong Nguyen Minh, Goutam Paul, Steven P. Harvey, Xiaopeng Zheng, Qi Jiang, Min Chen, Tobias Abzieher, Axel F. Palmstrom, Brian Habersberger, E. Ashley Gaulding, Joseph M. Luther, Lance M. Wheeler

**Affiliations:** † National Renewable Energy Laboratory, 15013 Denver West Parkway, Golden, Colorado 80401, United States; ‡ The Dow Chemical Company, 230 Abner Jackson Pkwy, Lake Jackson, Texas 77566, United States

**Keywords:** Perovskite photovoltaics, Encapsulation, Perovskite reliability, Vacuum lamination, Thermal degradation

## Abstract

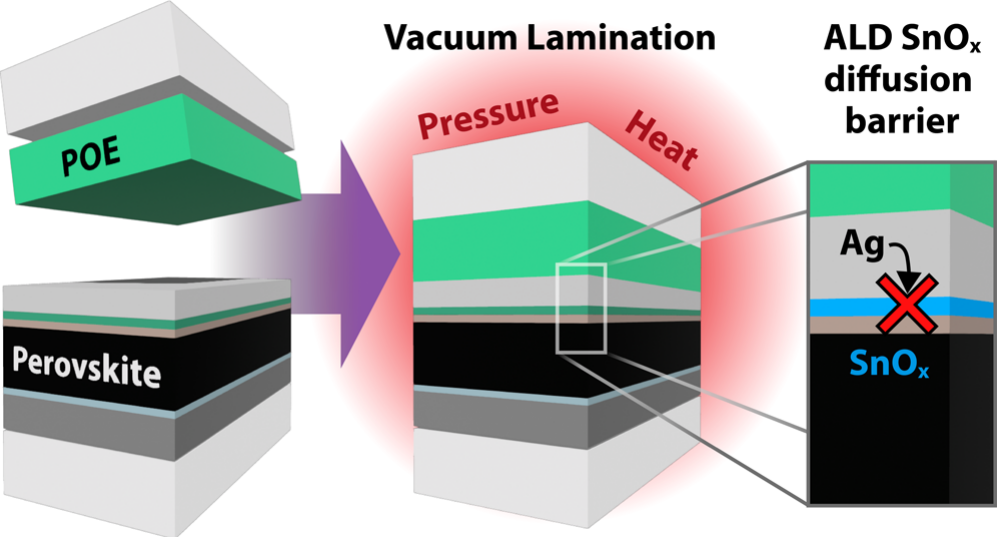

Current photovoltaic (PV) panels typically contain interconnected solar cells that are vacuum laminated with a polymer encapsulant between two pieces of glass or glass with a polymer backsheet. This packaging approach is ubiquitous in conventional photovoltaic technologies such as silicon and thin-film solar modules, contributing to thermal management, mechanical reinforcement, and environmental protection to enable the long lifetimes necessary to become financially acceptable. Commercial vacuum lamination processes typically occur at 150 °C to ensure cross-linking and/or glass bonding of the encapsulant to the glass and PV cells. Perovskite solar cells (PSCs) have emerged as a promising next-generation PV technology that is known to degrade under thermal stresses, especially at temperatures above 100 °C. In this study, we determine degradation modes during lamination and engineer internal diffusion barriers within the PSC to withstand the harsh thermal conditions of vacuum lamination. PSCs with self-assembled monolayers at the ITO interface and SnO_*X*_ layers deposited by atomic layer deposition at the electron extraction side of the device endured vacuum lamination at conditions typical of commercial PV processes (150 °C) without degradation. This work demonstrates that perovskite PV can be integrated into the existing module lamination process, enabling future single- and multijunction modules utilizing perovskite absorbers.

Perovskite solar cells (PSCs) are a promising development in the field of photovoltaics (PV), offering remarkable potential for high-efficiency and low-cost solar modules. Despite their promising performance, the commercialization and mass manufacturing of PSCs face critical challenges, primarily due to their sensitivity to humidity, temperature, oxygen, UV light, and mechanical stress.^1^ Ensuring mechanical robustness and impenetrable barrier layers is crucial for preserving the integrity and performance of these solar cells under diverse environmental conditions. Additionally, factors such as compatibility with existing manufacturing processes for tandem applications, cost-effectiveness, and long-term durability under outdoor operation are crucial considerations. It is essential to devise scalable manufacturing strategies and develop packaging techniques that address both the durability required for long-term outdoor operation and the operational demands of large-scale production. Although perovskites are commonly encapsulated with low-temperature-curing epoxy resin, which suffices for enhancing durability and shelf life under controlled indoor conditions, this approach is inadequate for real-world outdoor conditions.^2^

Vacuum lamination has been a cornerstone in the fabrication of silicon and thin-film solar modules, providing a low-cost and robust method for encapsulating solar cells to enhance their durability against outdoor exposure. However, vacuum lamination at conventional temperatures presents difficult challenges for PSCs due to the volatile organic cations of the absorber and reactive interfaces, particularly with metal electrodes.^3−9^ Cheacharoen et al. explored vacuum lamination strategies for PSCs, focusing on the testing of various polymeric encapsulants. Their research emphasized the importance of selecting polymers with a low elastic modulus to prevent delamination of the C_60_ layer, especially under thermal cycling and damp heat reliability testing.^10,11^ De Bastiani et al. determined C_60_ fractures at the interface to the SnO_*X*_ buffer layer.^12^ Baumann and colleagues investigated different lamination temperatures within the vacuum lamination process. They observed that devices may exhibit an immediate degradation in open-circuit voltage (*V*_OC_) following lamination, which then recovers after storage in nitrogen atmosphere and in the dark.^13^ Though individual parameters have been analyzed, most studies lack a comprehensive comparison of performance parameters for solar cells and modules before and after vacuum lamination. Furthermore, most studies focus solely on the investigation of individual material parameters rather than on a comprehensive exploration of variations in encapsulation, functional solar cell layers, and lamination processes.

In this work, we aim to bridge the gap between innovative perovskite technology and established PV fabrication processes, facilitating the integration of PSCs into existing manufacturing infrastructures. Our research systematically explores the effects of varying lamination process parameters, specifically temperature and duration, as well as the selection of materials for both the PSCs and the polymeric encapsulants. In the PSC, we vary the hole transport layer (HTL) and the buffer layer (BL) for the electron transport layer (ETL) to explore the durability during vacuum lamination. We determine that self-assembled monolayer (SAM) HTL materials such as [2-(9*H*-carbazol-9-yl)ethyl]phosphonic acid (2PACz), and [2-(3,6-dimethoxy-9*H*-carbazol-9-yl)ethyl]phosphonic acid (MeO-PACz) are more durable to lamination than poly[bis(4-phenyl)(2,4,6-trimethylphenyl)amine (PTAA).^14−16^ Tin oxide (SnO_*X*_) grown by atomic layer deposition (ALD) is more robust to lamination than widely used bathocuproine (BCP) as the ETL buffer layer.^17^ We propose silver (Ag) diffusion as the primary degradation mechanism during vacuum encapsulation by studying different permutations of PSC device materials and encapsulants and employing a variety of characterization techniques. We demonstrate that SAM HTLs coupled with a SnO_*X*_ ETL buffer layer as an internal diffusion barrier mitigate silver diffusion to facilitate vacuum lamination of PSCs at 150 °C—standard practice for the PV industry. Our research underscores the potential of PSCs to complement and enhance the current solar technology portfolio by enabling drop-in compatibility with existing manufacturing practices.

## Vacuum Lamination of PSCs

**Figure 1:**
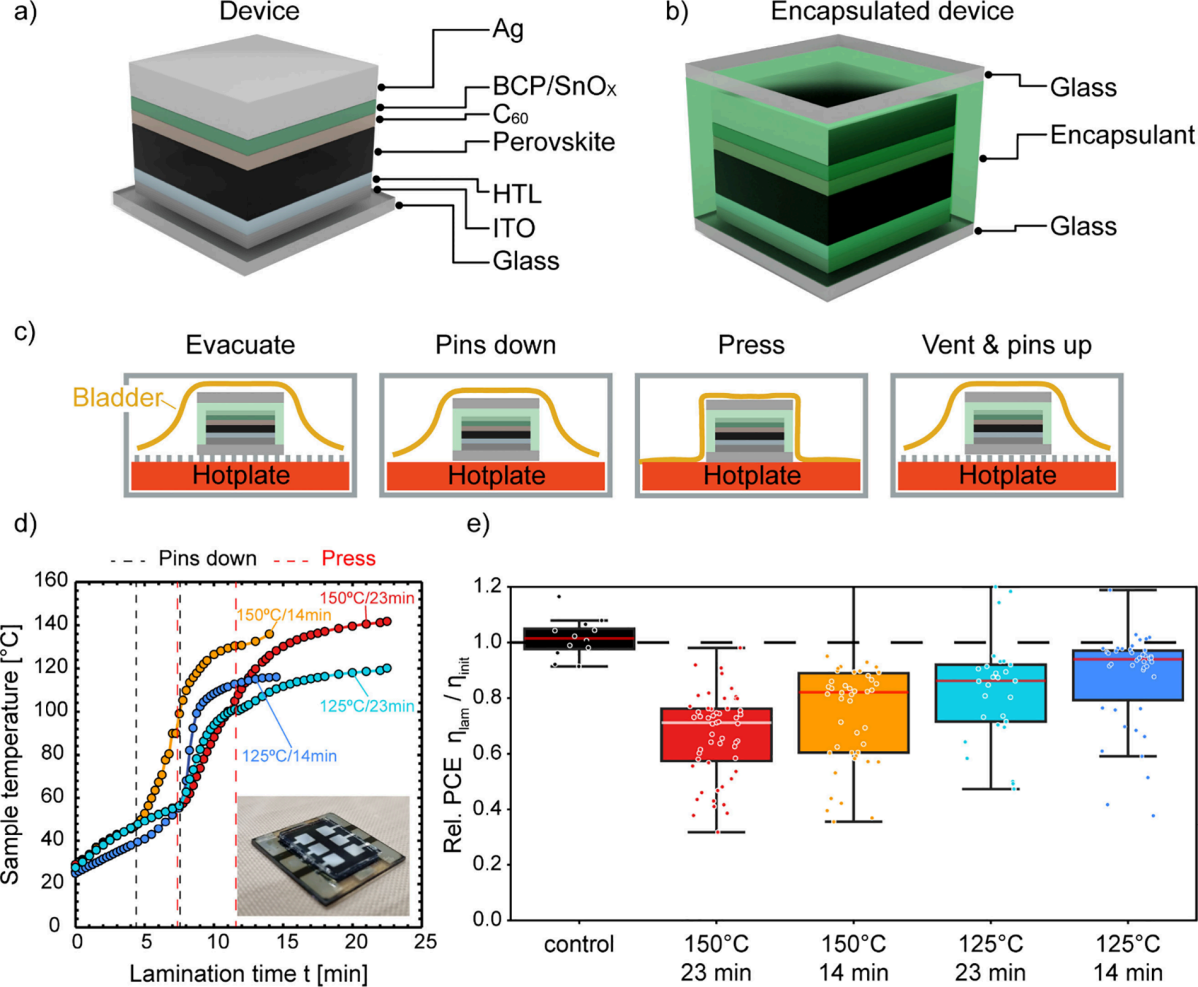
(a) Schematic of the device stack, highlighting the variations in the hole transport layer (HTL) and buffer layer (BL). (b) Schematic of the encapsulated device. (c) Illustration of the lamination process steps. (d) Sample temperature during the four lamination processes measured on the sample’s rear side. The evacuation starts immediately with the process. The dashed lines indicate time steps when the pins drop and the bladder starts pressing on the sample. Inset shows the six-pixel device after lamination. (e) Relative change in power conversion efficiency (PCE) for the reference group employing 2PACz as the HTL and BCP as the BL after laminating the samples with the four lamination processes. The control is an unencapsulated device to verify the reproducible measurement conditions.

We fabricated p-i-n PSCs with the device architecture depicted in Figure 1a (Methods, Supporting Information). In our study, we vary the HTL and BL to assess the durability of the PSC under various lamination conditions. A PSC with 2PACz as the HTL and BCP as the BL represents our baseline PSC.

We package these devices with a polymeric encapsulant and a rear cover glass (Figure 1b). For the encapsulation, we apply a vacuum lamination process using a pin vacuum laminator (Methods, Supporting Information). Figure 1c visualizes the lamination process. The device is placed in the laminator on pins with the substrate glass facing the laminator hotplate and a stack of extruded polymeric encapsulant and cover glass on the device’s rear side. All baseline PSCs employ silane-grafted polyolefin elastomers (POEs) with low crystallinity (Table S1). During the lamination process, the sample initially undergoes a degassing phase, where the lamination chamber is evacuated. In this phase, the device is positioned on pins to elevate it above the hotplate, which is preheated to the specified set temperature. Following this, the pins retract, allowing the substrate glass to directly contact the hotplate for heating. Subsequently, the bladder gradually applies pressure to the sample, increasing by 7 kPa every 15 s until reaching a maximum pressure of 81 kPa.

We employ four lamination profiles with different set temperatures and process durations. Figure 1d shows the sample temperature profile during each lamination process measured on the cover glass on the rear side of the sample. The labels indicate the set temperature and process duration for each lamination process. The dashed lines in Figure 1d indicate the times when the pins retract and the bladder begins to apply pressure to the device. The most thermally intensive process of 150 °C for 23 min is akin to conventional conditions used for silicon or thin-film module lamination. We then systematically decrease the temperature and time to reduce the thermal stress on the PSCs. A minimum lamination temperature of 125 °C is used since temperatures below this threshold lead to reduced polymer adhesion to the glass and insufficient bonding, resulting in mechanically unstable devices. Throughout the manuscript, we denote these lamination processes as 150 °C/23 min, 150 °C/14 min, 125 °C/23 min, and 125 °C/14 min.

We conduct comprehensive pre- and post-lamination analyses employing current density–voltage (*JV*) measurements, spatial electroluminescence (EL) and photoluminescence (PL) imaging, cross-sectional Kelvin probe force microscopy (cKPFM),^18^ X-ray diffraction (XRD), and time-of-flight secondary ion mass spectrometry (ToF-SIMS)^19^ measurements to evaluate the impact of different lamination conditions on the various device architectures (for detailed methods, see the Supporting Information). It is important to note that we observe a recovery in *JV* parameters when measuring the devices immediately after lamination, which stabilize after storing in the dark in a nitrogen atmosphere for several hours (Figure S1).^13^ Hence, all reported values in the following section pertain to measurements taken after resting the devices for at least 2 days in the dark in a nitrogen atmosphere.

## Effect of the Lamination Conditions on the Baseline PSC

Figure 1e shows the relative change in the power conversion efficiency (PCE) of the baseline PSC group before and after lamination with the four lamination processes. Our control is an unencapsulated device to verify reproducible measurement settings. We find that a higher process temperature and duration results in higher performance loss of the PSC after the lamination process. Samples laminated with the 150 °C/23 min process show the highest average loss in PCE of 27%_rel_. Samples exhibiting significant degradation also show the development of hysteresis. The degradation presented in Figure 1e is calculated as the average of the forward and reverse *JV* sweeps, thereby reflecting the combined degradation of both characteristics (Figure S2). The lowest average loss of 6%_rel_ we measure for the group laminated with the 125 °C/14 min process. The loss in PCE correlates with a reduction and higher variance in short-circuit current density (*J*_SC_) and fill factor (FF), while the open-circuit voltage (*V*_OC_) is more stable and shows less variance (Figure S3). For instance, we observe an average loss of 10% in *J*_SC_ and 15% in FF, while the loss in *V*_OC_ is only 7% when laminating at 150 °C for 23 min. This is in agreement with other studies reporting on thermo-compressive stress on PSCs.^20^

Initially, we hypothesized that the viscosity and linking chemistry of the polymeric encapsulant will lead to mechanical or chemical degradation modes during the lamination process. We used six different polymeric encapsulants, each varying in linking chemistry, melting temperature, and elastic modulus (Table S1). We utilized conventional peroxide-cured ethylene vinyl acetate (EVA) and POE, which are cross-linking encapsulants used in silicon solar modules. These were compared with custom-synthesized POEs grafted with silane and anhydride chemistries, which yield different linkage chemistries at the device interface and different reaction byproducts during lamination.^21,22^ All polymeric encapsulants have low material shrinkage to reduce the mechanical stress created during lamination.^23^ We also developed low- and high-crystallinity versions of the POEs to independently tailor the physical properties of the polymers. Low-crystallinity POEs have a lower elastic modulus, which has been reported to be beneficial for perovskite encapsulation.^11^ In contrast, in our study, the variation of the polymeric encapsulant has no significant effect on the device performance after lamination (Figure S4). Cheacharoen et al. reported that using polymers with lower elastic moduli improves the lamination durability of PSC.^10^ Yet, their comparison involved polymers with elastic moduli of 394 MPa and 7 MPa, a significantly broader range than that explored in our study. Moreover, our investigations focus on single PSC without interconnection by scribe lines, and thus, the perovskite absorber is isolated from direct contact with the polymeric encapsulant. Hence, we cannot dismiss the possibility that the linking chemistry could influence PSCs with scribe lines, where these layers directly interface with the polymer. Additionally, some potentially damaging phenomena that may occur during extended weathering, such as the generation of acetic acid by EVA, are not factors in the experiments here, which focus on the lamination performance.

## Effect of Lamination on PSCs with Varying HTL and BL

**Figure 2:**
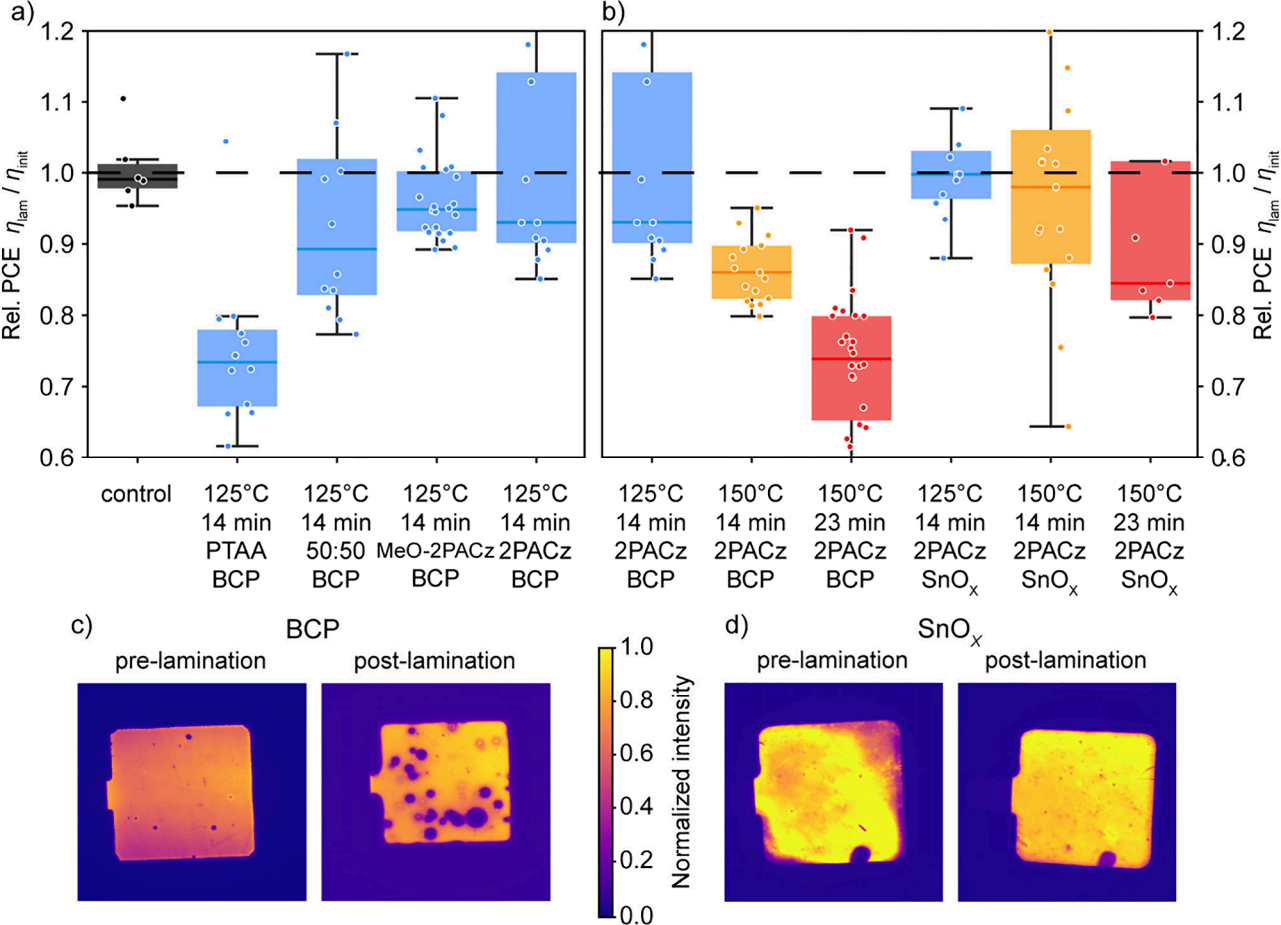
(a, b) Relative change in power conversion efficiency (PCE) before and after lamination for devices with (a) varying hole transport layer (HTL) and (b) varying buffer layer (BL) laminated with the 125 °C/14 min, 150 °C/14 min, and 150 °C/23 min processes. (c, d) Electroluminescence images at short-circuit conditions of devices pre- and post-lamination with the 150 °C/14 min process using different buffer layers of (c) BCP or (d) SnO_*X*_.

Beyond the baseline PSC, we apply SnO_*X*_ via atomic layer deposition (ALD) as an alternative BL to BCP and replace 2PACz with PTAA, MeO-2PACz, and a 50:50 mixture of MeO-2PACz and 2PACz as HTL materials. Figure 2a shows the relative changes in PCE for devices with varying HTL materials for different lamination conditions. At 125 °C/14 min lamination conditions, devices with PTAA and the 50:50 mix of MeO-2PAC:2PACz show the highest PCE losses of 27%_rel_ and 11%_rel_, respectively. Even under the least thermally intensive lamination process, significant degradation is observed. Samples with MeO-2PACz and 2PACz show the lowest average loss of about 6%_rel_, which is consistent with the data in Figure 1e for our baseline device. We hypothesize that the SAM layers are advantageous due to their better mechanical robustness,^14^ which is beneficial in withstanding the thermomechanical stress during lamination.

Figure 2b shows the relative change in PCE for devices with varying BL materials for different lamination conditions. Using SnO_*X*_ as the buffer layer in place of BCP enhances the PSC durability under vacuum lamination. Devices with 2PACz as the HTL and SnO_*X*_ as the BL exhibit no degradation within the limits of measurement uncertainty following the 125 °C/14 min lamination process and only 3%_rel_ degradation on average after the 150 °C/14 min process.

The lamination process clearly damages the perovskite absorber layer when using the baseline device stack. Figure 2c shows EL images under short-circuit conditions of a baseline device, featuring 2PACz as the HTL and BCP as the BL, pre- and post-lamination with the 150 °C/14 min process. After lamination, distinct circular areas without luminescence become visible, either due to shunting or increased nonradiative recombination.^24,25^ This observation is associated with a reduction in the photoactive area and shunt resistance, correlating with the decreases in *J*_SC_ and FF from the *JV* measurements (Figure S3). We assume the shunts could be formed by the penetrating Ag or the ion redistribution.^26^ Additionally, numerous EL and PL images of devices with a BCP BL exhibit “edge effects” after lamination (Table S2), which are attributed to lateral ion migration and accumulation at the perimeter of the device’s active area.^27^ We exclude any effects of phase segregation^28,29^ or silver-halide formation,^30^ as no corresponding changes are observed in the XRD patterns (Figure S5).

Figure 2d shows EL images under short-circuit conditions of a device, featuring 2PACz as the HTL and SnO_*X*_ as the BL, before and after lamination with the 150 °C/14 min process. In comparison to the baseline device in Figure 2c, reduced post-lamination degradation is evident for devices featuring the SnO_*X*_ buffer layer, with no regions showing diminished luminescence. Additional EL and PL images of devices with a SnO_*X*_ BL before and after lamination are provided in Table S3. Only upon escalating the process temperature and duration to the 150 °C/23 min lamination process do we observe an average degradation of 16%_rel_ for these devices.

From these findings, we infer that specifically utilizing 2PACz or MeO-2PACz as the HTL and ALD SnO_*X*_ buffer layers significantly enhances the vacuum lamination durability of the PSC, approaching the conditions typical for packaging of conventional silicon or other thin-film technologies.

## Degradation Mechanisms during Vacuum Lamination

**Figure 3:**
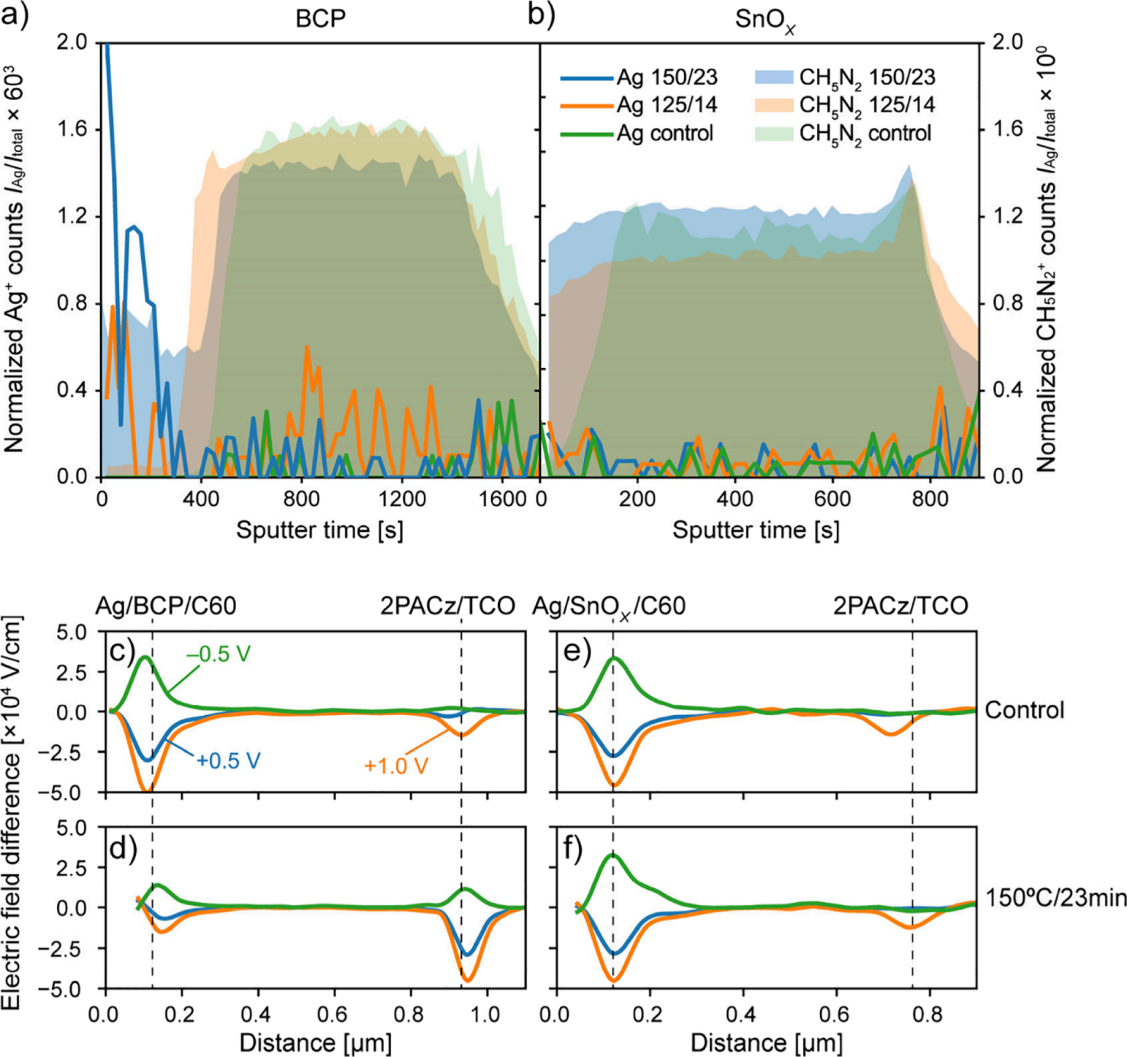
Depth profiles of Ag and CH_5_N_2_ from time-of-flight secondary ion mass spectrometry (ToF-SIMS) measurements for devices using (a) BCP or (b) SnO_*X*_ as buffer layers. Each profile compares nonlaminated control devices to those laminated with the 125 °C/14 min and 150 °C/23 min processes. (c–f) External bias voltage induced electric field difference (EFD) derived from cross-sectional Kelvin probe force microscopy (cKPFM) measurements of nonlaminated control devices and devices laminated with the 150 °C/23 min process incorporating either (c, d) BCP or (e, f) SnO_*X*_ as buffer layers.

We investigate mechanisms of degradation by correlating time-of-flight secondary ion mass spectrometry (ToF-SIMS)^19^ chemical analysis with cross-sectional Kelvin probe force microscopy (cKPFM)^18^ to observe chemical and electrical changes in the device operation after lamination. Figure 3a shows depth profiles of Ag and CH_5_N_2_ species from ToF-SIMS measurements on PSC devices with BCP as the buffer layers. The CH_5_N_2_ signal identified the perovskite absorber. We compare nonlaminated control devices to those laminated with the 125 °C/14 min and 150 °C/23 min processes. Figure 3b shows the same measurements for devices featuring a SnO_*X*_ BL. All samples utilize 2PACz as the HTL. In Figure 3a, there is an increase in Ag signal at the ETL interface and across the active layer for samples with BCP buffer layers. The amount of Ag increases with increasing temperature and duration in the lamination process.

In contrast, for the samples with SnO_*X*_ as the BL in Figure 3b, we observe no increase in the Ag signal at the ETL side and across the sample.

Figure 3c,d shows the external bias voltage induced electric field difference (EFD) derived from cKPFM measurements of a nonlaminated control device and a device laminated with the 150 °C/23 min process, incorporating BCP as the buffer layer. Figure 3e,f shows the same measurements for devices with SnO_*X*_ as the BL. Both samples utilize 2PACz as the HTL. We apply bias voltages of −0.5 V, 0.0 V, 0.5 V, and 1.0 V for the cKPFM measurements.

The nonlaminated control sample with a BCP BL in Figure 3c shows a higher amplitude in the EFD signal at the ETL interface relative to the HTL interface. This higher amplitude in the EFD signal suggests either an elevated equivalent resistance at the ETL interface or an enhanced conductance at the HTL interface.^18,31^ The control device and the device laminated with the 150 °C/23 min process with SnO_*X*_ BLs in Figure 3e,f show only marginal differences in the EFD signal, indicating that the interface is unaffected by the lamination process. However, for the device laminated at 150 °C/23 min with a BCP buffer layer in Figure 3d, a notable reversal occurs in the EFD signal intensity, which is now higher at the HTL interface compared to the ETL interface. The electric field drops more at the HTL interface, suggesting either an increased equivalent resistance at the HTL interface or an enhanced conductance at the ETL interface after the lamination process. The internal electric field in the device has fundamentally changed after lamination.

Based on our observations in the cKPFM and ToF-SIMS measurements, we hypothesize that ALD SnO_*X*_ is a more effective diffusion barrier against Ag migrating from the Ag metal contact, thereby preventing Ag from infiltrating the perovskite absorber during lamination. The SnO_*X*_ barrier also prevents halides from migrating to the Ag electrode, where irreversible reactions lead to silver-halide formation.^3,7,30^

Conversely, BCP is a poor diffusion barrier, allowing Ag to migrate from the metal contact into the perovskite absorber during lamination. The higher the temperature and the duration during the lamination process, the greater the extent of Ag diffusion into the perovskite, which degrades the device’s performance. Zhang et al. have similarly reported on this phenomenon of electrode metals penetrating the C_60_ layer.^3^ The incorporation of Ag enhances the ETL interface conductivity, which aligns with our observations in the cKPFM measurement. We hypothesize that the heat during the lamination process promotes Ag diffusion from the contact into the ETL and perovskite absorber. This diffusion can occur either as metallic silver (Ag^0^), which forms shunt paths, observable in reduced current and luminescence imaging, or as Ag^+^ ions, which occupy interstitial sites within the absorber, leading to electrochemical doping.^32^ Both mechanisms are detrimental to the device performance and contribute to degradation.

## Conclusions

Our investigations into the effects of vacuum lamination on perovskite solar cells (PSCs) reveal that the temperature and duration of the lamination process significantly impact the PSC performance. Specifically, we observe that higher process temperatures and durations correlate with increased performance loss, with the 150 °C/23 min lamination condition exhibiting the most substantial decrease in PCE, primarily due to reductions in *J*_SC_ and FF. Conversely, *V*_OC_ is less affected, suggesting that lamination primarily impacts the interfaces between the perovskite and transport layers rather than the perovskite itself.

Based on the variations observed in the HTL, our findings indicate that self-assembled monolayers such as 2PACz or MeO-2PACz offer advantages over PTAA. However, it is important to highlight that our study did not include an examination of NiO_*X*_. Given the superior thermal stability of NiO_*X*_, it might offer comparable durability benefits.

The most important finding is that BCP as a buffer layer is less suitable than SnO_*X*_ for improving the lamination durability of PSC. Besides *JV* characterization, EL imaging confirms the excellent lamination durability of devices with SnO_*X*_ buffer layers, demonstrating minimal degradation even under lamination at 150 °C. This resilience, combined with observed durability improvements when employing 2PACz or MeO-2PACz as the HTL, suggests a pathway to enhancing PSC lamination durability toward processes employed in established PV manufacturing technologies.

After further investigating the underlying degradation mechanism through cKPFM and ToF-SIMS measurements, we hypothesize that SnO_*X*_ is a more effective barrier against Ag diffusion into the perovskite absorber than BCP.

Our comprehensive study bridges the gap in understanding the complex interplay among lamination conditions, material choices, and the subsequent effects on PSC lamination durability and performance. It offers valuable insights for refining encapsulation techniques to seamlessly integrate perovskite single-junction and tandem solar cells within the current photovoltaic production framework and for enhancing the long-term reliability of PSCs under outdoor operation.

## Supporting Information Available

Experimental methods; polymeric encapsulants overview; temporal post-lamination device stability; representative *JV* curves; relative change in *J*_SC_, *V*_OC_, and FF; and EL and PL images of devices pre- and post-lamination (PDF)
