# Supplementary material for: Reducing Thermal Degradation of Perovskite Solar Cells during Vacuum Lamination by Internal Diffusion Barriers
Source: ACS Appl Energy Mater. 2024 Nov 7;7(22):10750–7. doi: 10.1021/acsaem.4c02567 (PMC11600410; doi:10.1021/acsaem.4c02567)
Supplement: Supplementary file 1 — ae4c02567_si_001.pdf [file ae4c02567_si_001.pdf]

Supporting Information

Reducing Thermal Degradation of Perovskite Solar Cells During Vacuum Lamination by Internal Diffusion Barriers

Robert Witteck[1*], Duong Nguyen Minh[1], Goutam Paul[1], Steven P. Harvey[1], Xiaopeng Zheng[1], Qi Jiang[1], Min Chen[1], Tobias Abzieher[1], Axel F. Palmstrom[1], Brian Habersberger[2], E. Ashley Gaulding[1], Joseph M. Luther[1], and Lance M. Wheeler[1*].

1. National Renewable Energy Laboratory, 15013 Denver West Parkway, Golden, CO, 80401 USA
2. Dow Chemical Company, 230 Abner Jackson Pkwy, Lake Jackson, TX, 77566 USA

*Corresponding Authors: Robert Witteck (robert.witteck@nrel.gov) and Lance M. Wheeler (lance.wheeler@nrel.gov)

Experimental Methods

Device fabrication

PbI_2 (507 mg), $PbBr_2$ (73.4 mg), MABr (22.4 mg), FAI (172 mg), and CsI (15.6 mg) were dissolved in a 1 ml mixture of DMF and DMSO (4:1 volume ratio) and vortexed to create a 1.3 M solution. This solution was then filtered using a 0.22 µm PTFE filter. Patterned ITO substrates (15 Ω/sq) were cleaned with Liquinox detergent, followed by sonication in deionized water (15 min), acetone (15 min), and isopropanol (15 min). The substrates were dried with nitrogen and treated with UV ozone for 15 minutes. A hole transport layer was spin-coated onto the substrates at 3000 rpm for 30 seconds in a nitrogen glovebox and then annealed at 100°C for 10 minutes. Next, 120 µL of the perovskite solution was spin-coated at 3500 rpm for 40 seconds. During the last 5-7 seconds of spin-coating, 150 µL of chlorobenzene was applied to the film as an antisolvent. Then, 25 nm of C60 was thermally evaporated onto the perovskite film at a rate of 0.2-0.5 Å/s. For the baseline samples, 3 nm of BCP was evaporated afterwards. For the SnO_X buffer layer, 20 nm of tin oxide (SnO_X) was deposited using a Beneq TFS-200 ALD system. Finally, 100 nm of silver (Ag) was deposited by thermal evaporation.

Encapsulant synthesis

Peroxide-crosslinking encapsulants (EVA-X and POE-L-X) were obtained from a commercial manufacturer. The remainder of the encapsulants are ethylene/alpha-olefin copolymers that have been reactively extruded with anhydride or alkoxysilane functionality to produce a modified resin; this category of materials is often referred to as “thermoplastic polyolefin” or TPO by the photovoltaic industry, although this terminology is confusing and we avoid using it in this report. Unlike peroxide-crosslinking encapsulants where significant crosslinking takes place during lamination, grafted polyolefins (and additional encapsulant solutions referred to as TPO but based on different chemistries) experience minimal change in molecular weight during lamination, but may subsequently crosslink through slower processes while in use.[1]

Device vacuum lamination

We use a conventional pin vacuum laminator (Bent river SPL2828 PIN Solar Panel Laminator) to encapsulate these devices. Each device is positioned in the laminator with substrate glass facing the hotplate, while a stack of encapsulation polymer and glass covers the rear side. The lamination process consists of a degassing, heating, incremental pressure, maximum pressure, and venting phase. During the degassing phase the chamber is evacuated to remove air and any trapped gases from between the layers. In this phase, the device is placed on pins to elevate it above the preheated hotplate set to 150°C. The pins then retract, allowing direct contact between the substrate glass and the hotplate for heating. For the 14 min process the pins retract after 255 s and for the 23 min process after 458 s. Subsequently, the bladder applies pressure to the sample, gradually increasing by 7 kPa increments until reaching a maximum pressure of 81 kPa. The pressing starts for the 14 min process after 420 s and for the 23 min process after 705 s. This maximum pressure is maintained for 6 minutes before the lamination chamber is vented, completing the process.

Solar cell characterization

The solar cells were measured under standard testing conditions (STC) using a G2V SunBrick AAA LED solar simulator. All solar cells were masked to an area of 0.0587 cm^2. The spectrum was calibrated with a Si reference cell and KG2 filter certified by NREL’s certification and measurement group. All solar cells were measured from –0.2 V to 1.2 V (forward direction) and from 1.2 V to –0.2 V (reverse direction) with a scan-rate of 0.44074 V/s. For the evaluation of the data before and after lamination we take the average *JV* of forward and reverse direction.

Photoluminescence (PL) imaging

Photoluminescence imaging was conducted using a 532 nm laser and galvanometer scanners to uniformly illuminate the active area of each device. The laser intensity was adjusted to simulate 1 sun conditions. Each device was measured under open-circuit conditions and illuminated for 2 minutes to achieve durability before capturing each image. Images were captured using a Princeton Instruments PIXIS Silicon CCD camera with 715 nm long pass filter.

Electroluminescence (EL) imaging

Electroluminescence imaging was conducted immediately after photoluminescence imaging using a Princeton Instruments PIXIS Silicon CCD camera equipped with a 715 nm long-pass filter. During imaging, the device was forward biased to match operating conditions of 0.1× Jsc and 1×Jsc

Time-of-flight secondary ion mass spectroscopy (ToF-SIMS)

TOF-SIMS measurements were carried out with a three-lens 30 keV BiMn primary ion gun. For high-mass-resolution depth profiling, a 30 keV Bi3+ primary ion beam with a pulsed beam current of 0.8 pA was used to analyze a 50 µm × 50 µm area, sputtering a 130 µm × 130 µm area. The device's Ag contact was delaminated using tape prior to the TOF-SIMS measurements.

Cross-sectional Kelvin probe microscopy (c-KPFM)

A home-built c-KPFM set-up inside an Ar-filled glove box was employed. We used a Pt/Ir-coated silicon probe with the tip's apex radius less than 25 nm for KPFM measurement. In our set-up, we selected the first resonant oscillation of the cantilever (50–70 kHz) for the AFM topographic imaging and the second resonant frequency (300–500 kHz) for the surface potential imaging. The spatial and voltage resolutions of our KPFM setup are ~30 nm and ~10 mV, respectively. To prepare a cross-section, devices were mechanically cleaved inside the Ar-filled glove box, and the KPFM measurement was done on a clean and smooth region of the device cross-section. We didn't perform any polishing or ion-milling treatment on the device cross-section to avoid artifacts induced during sample preparation. For voltage bias induced measurements, the Ag back-contact was grounded, and a bias voltage (Vb) applied to the ITO. To obtain the external bias voltage induced electric field distribution across the device stack, we recorded the surface potential images under different bias voltages: Vb = 0V, +0.5V, +1V (forward bias), and -0.5V (reverse bias). The scan rate was 0.3 Hz. Then, we obtained the potential profiles across the device cross-section by averaging the lines (32 lines) in the potential image over a few hundred nanometers. We subtract the 0V potential profile from the other profiles under different bias voltages (Vb) to derive the potential difference profiles. This potential difference profile is related to the drop of external bias voltage across the device cross-section. Finally, the electric field difference profile was derived by taking the first derivative of the potential difference profile.

X-Ray Diffraction (XRD)

X-ray diffraction (XRD) measurements were conducted using a Bruker D8 Discover system equipped with a Cu K-alpha radiation source and an area detector.

Table S1 Investigated polymeric encapsulants. For the packaging, we employ six different polymeric encapsulants, each varying in linking chemistry, melting temperature, and elastic moduli. Peroxide-cured ethylene-vinyl acetate (EVA-X) and polyolefin elastomers (POE-X) are similar to the encapsulation materials employed in conventional silicon modules. Additionally, we utilize POEs with silane- (Si) and anhydride- (Ah) grafted chemistries, which result in a different interaction with the device surface during lamination due to the introduction of new functional groups.[2,3] Furthermore, we employ low (L) and high (H) crystalline versions of POEs, tailoring the physical properties of the polymers. The low crystalline POEs have a lower elastic modulus, which has been reported to be beneficial for perovskite encapsulation.[4]

Encapsulation polymer	Formulation	Melting temperature [°C]	Elastic modulus [MPa]
EVA-X	Peroxide-crosslinking	70	10-15
POE-L-X	Peroxide-crosslinking	72	10-15
POE-H-Si	Silane-grafted	95	20-30
POE-L-Si	Silane-grafted	72	10-15
POE-H-Ah	Anhydride-grafted	95	20-30
POE-L-Ah	Anhydride-grafted	72	10-15

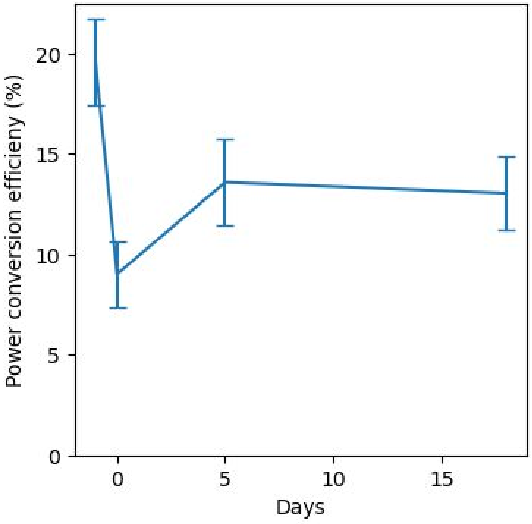

Figure S1: Power conversion efficiency (PCE) of devices measured before lamination (-1 days), within 1h, 5 days, and 18 days after lamination with the 150°C/23 min process.

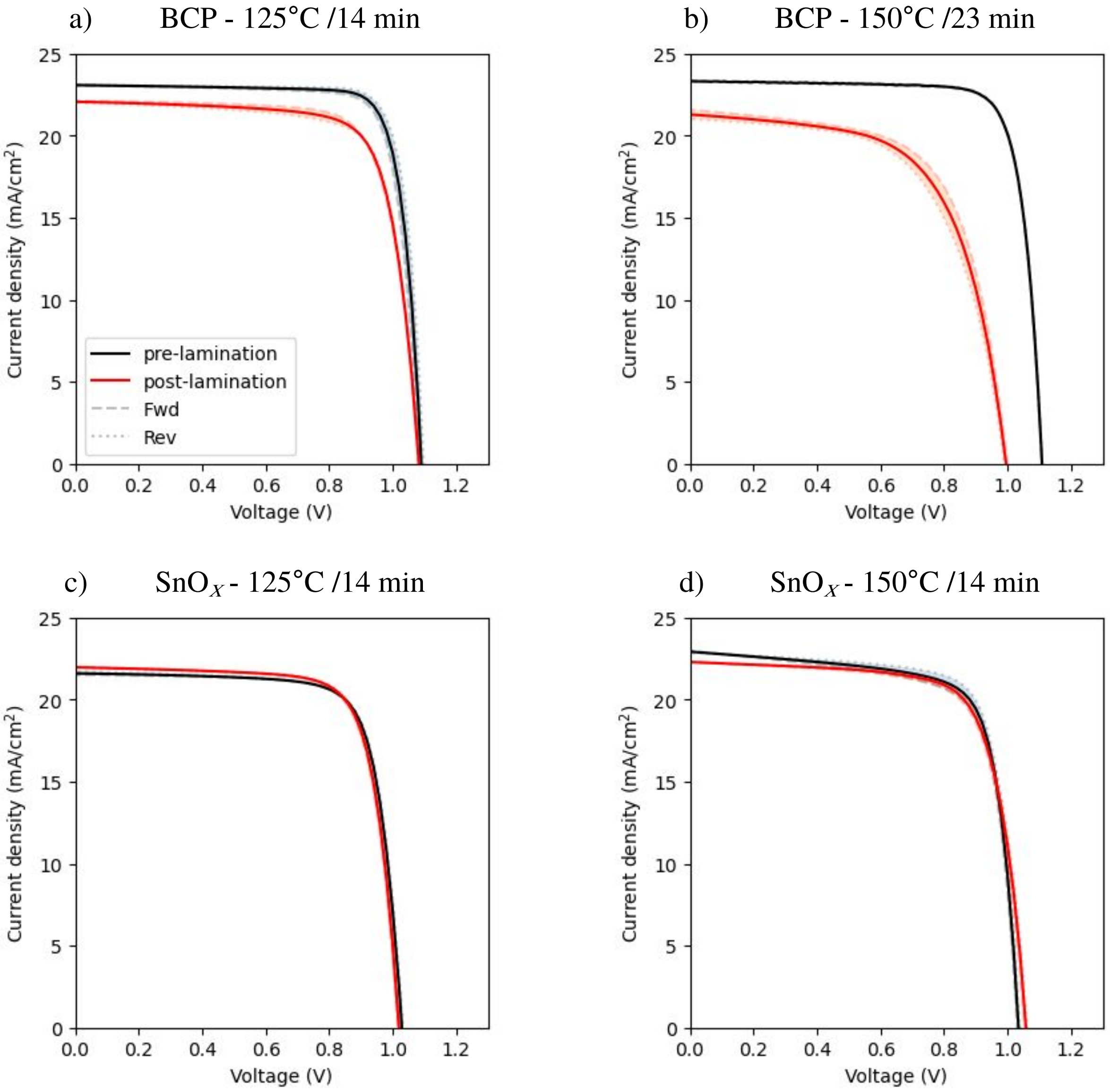

Figure S2: Representative *JV* curves of samples with a) BCP laminated at 125°C /14 min, b) BCP laminated at 150°C /23 min, c) SnO_X laminated at 125°C /14 min, and d) SnO_X laminated at 150°C /14 min process.

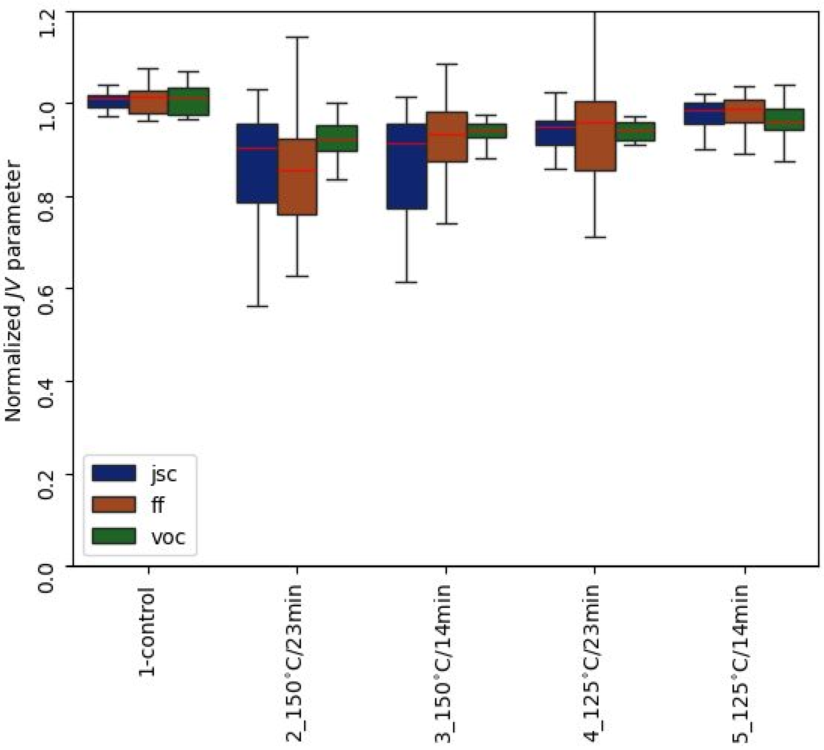

Figure S3: Relative change in J_{SC}, FF, and V_{OC} for samples laminated with various lamination conditions.

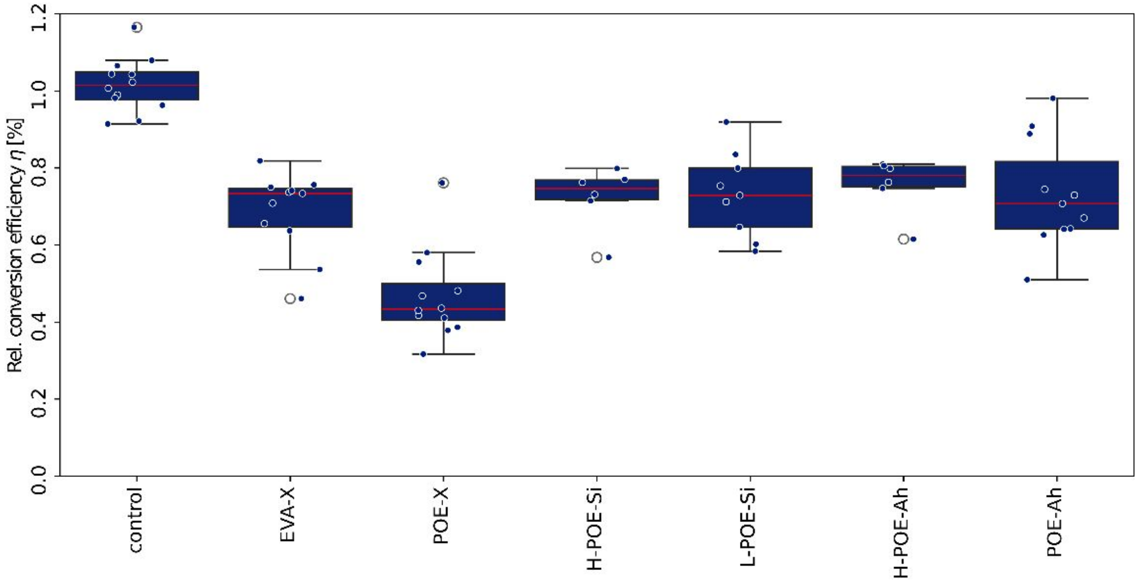

Figure S4: Samples laminated with the 150°C/23min lamination process employing various polymeric encapsulants. Besides the POE-X we observe a similar loss for all groups using the various polymeric encapsulants.

**Table S2: Example EL and PL images of devices with BCP buffer layer pre- and post-lamination.**		
Lamination	EL	PL
BCP Device 1<br>Pre-lamination	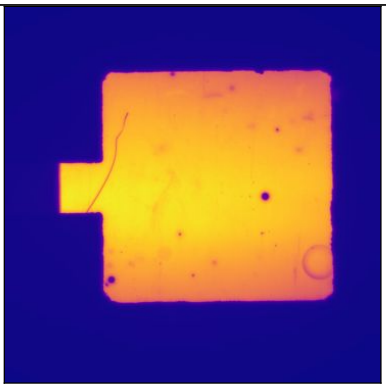	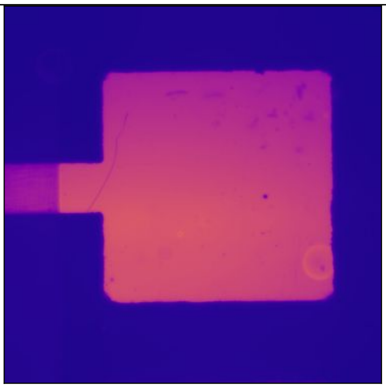
BCP Device 1<br>Post-lamination	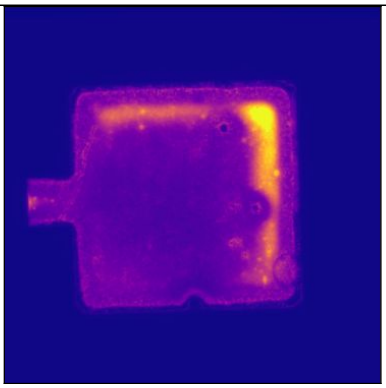	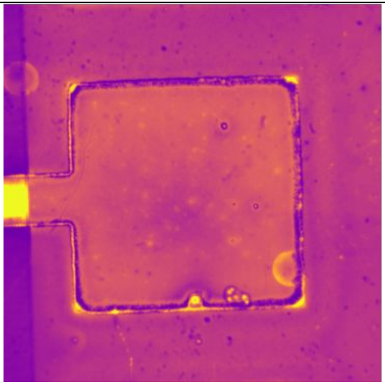
BCP Device 2<br>Pre-lamination	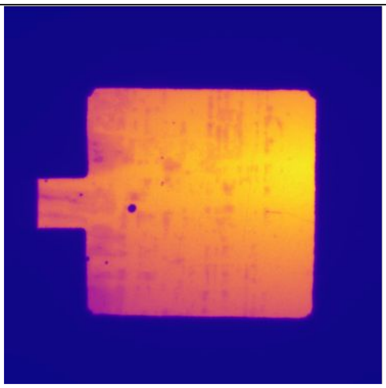	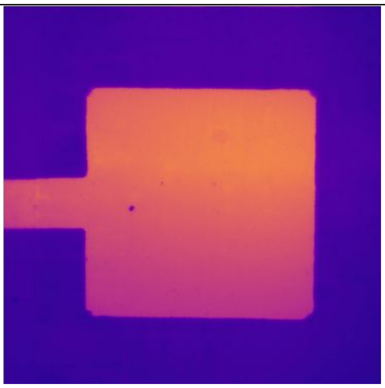
Device 2<br>Post-lamination	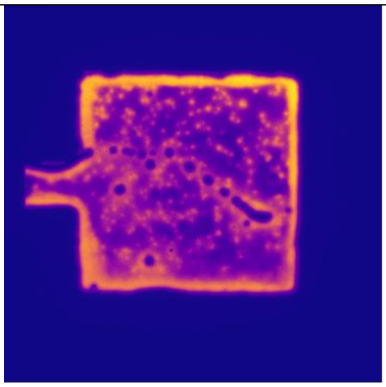	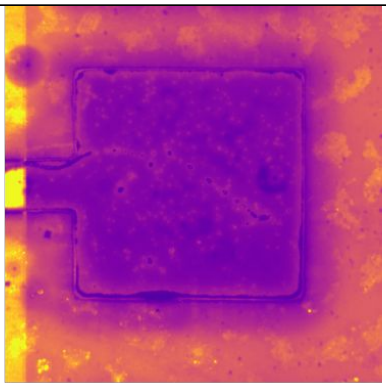

Device 3<br>Pre-lamination	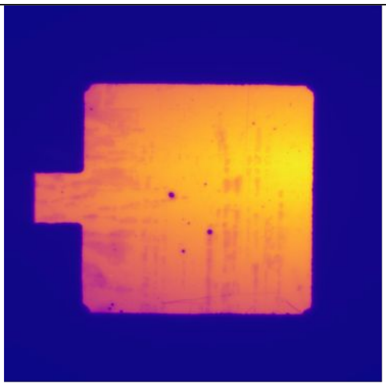	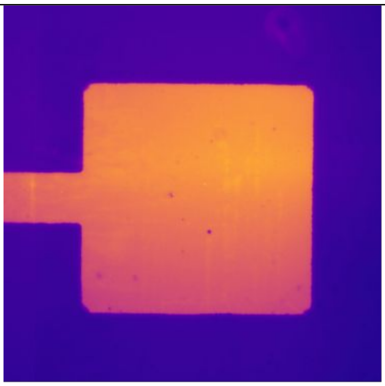
Device 3<br>Post-lamination	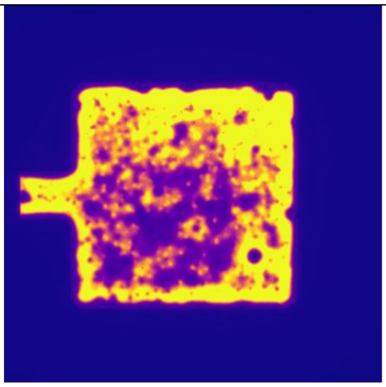	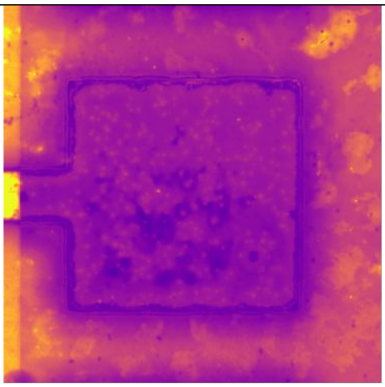
Device 4<br>Pre-lamination	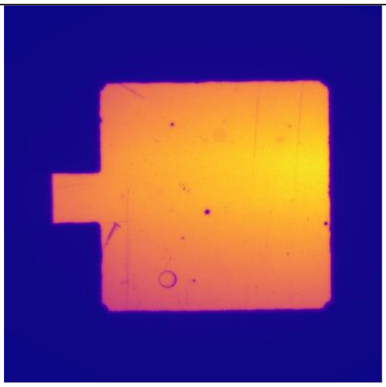	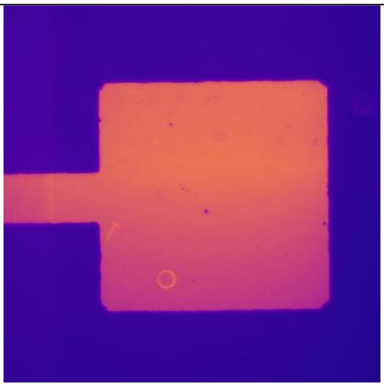
Device 4<br>Post-lamination	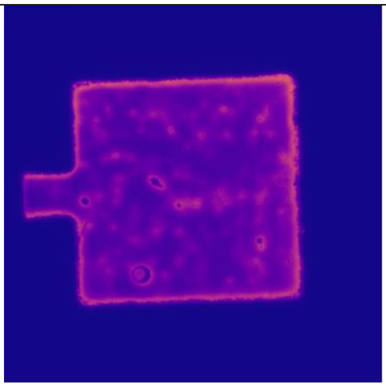	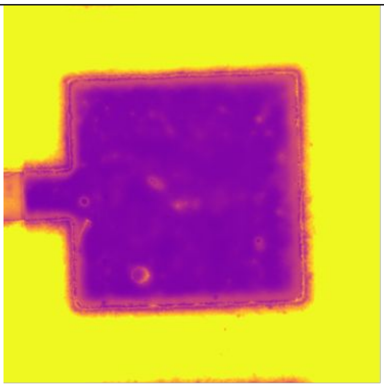

Device 5<br>Pre-lamination	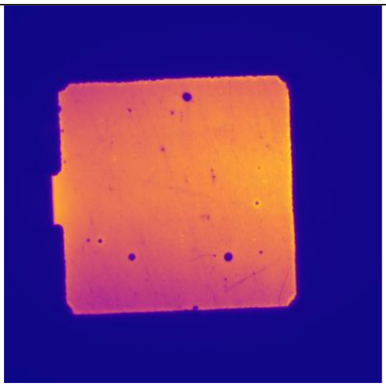	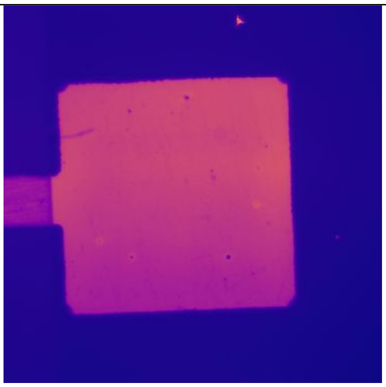
Device 5<br>Post -lamination	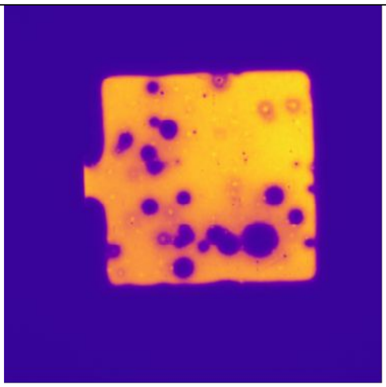	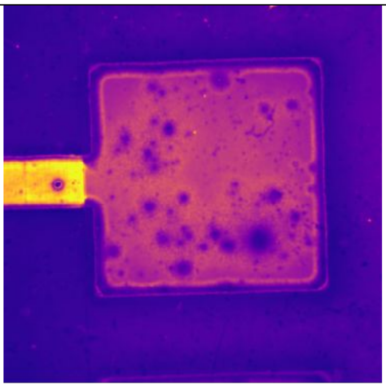

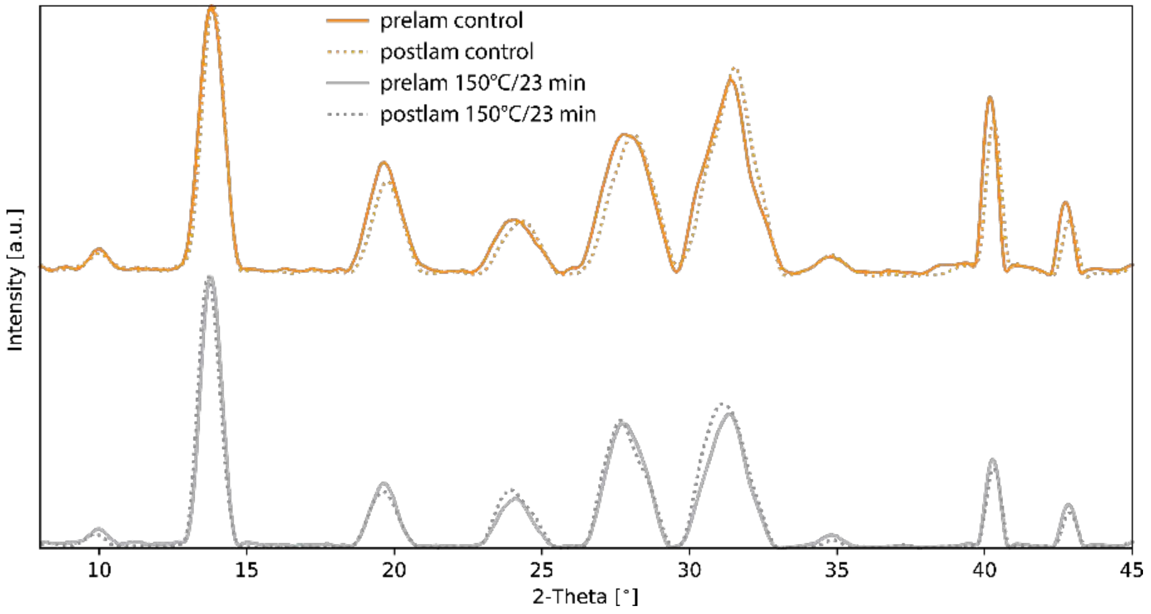

Figure S5: XRD pattern of control sample (not laminated) and a sample before and after lamination with the 150C/23 min process.

Table S3: Example EL and PL images of devices with SnO_X buffer layer pre- and post-lamination.

Lamination	EL	PL
SnO_X device 1 Pre-lamination	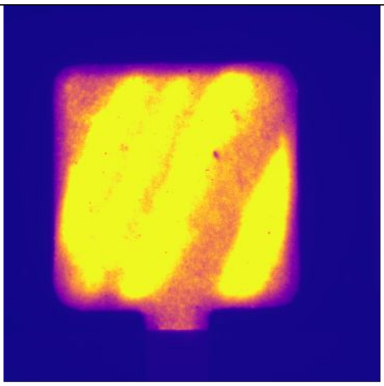	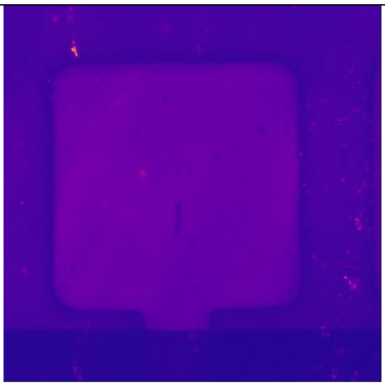
SnO_X device 1 Post-lamination	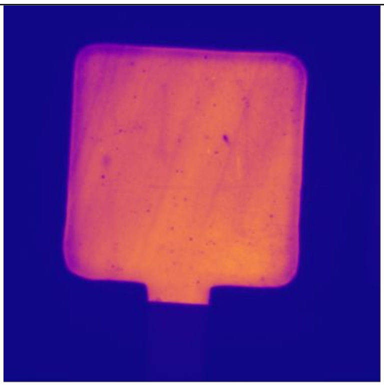	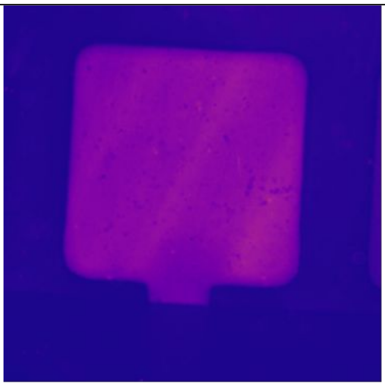
SnO_X device 2 Pre-lamination	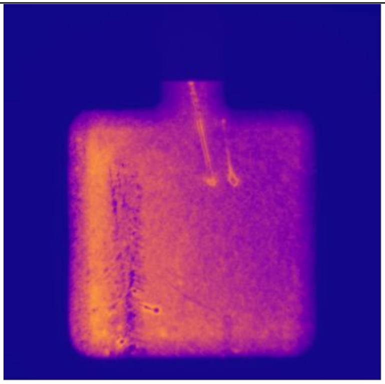	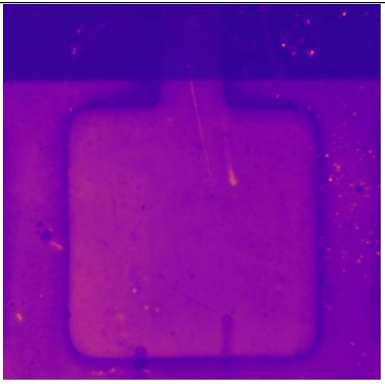
SnO_X device 2 Post-lamination	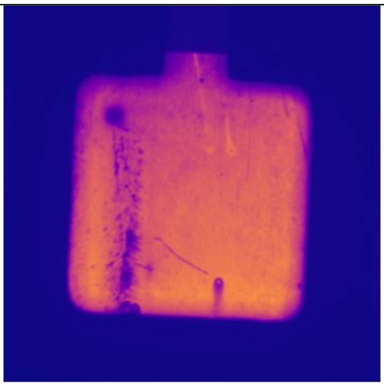	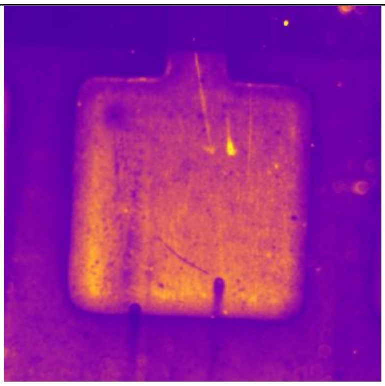

SnO_x device 3<br>Pre-lamination	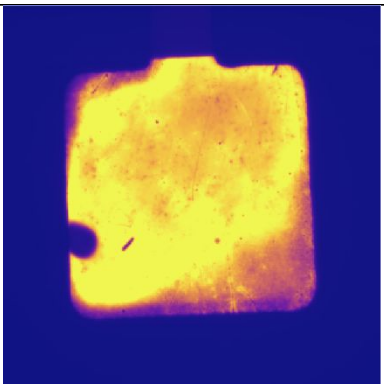	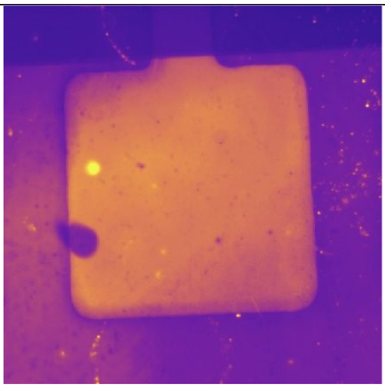
SnO_x device 3<br>Post-lamination	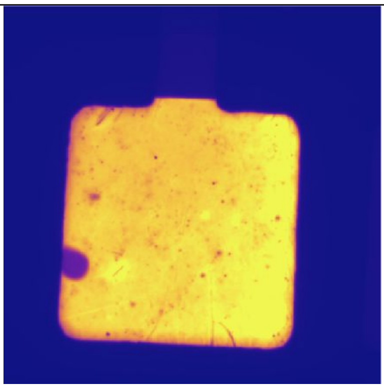	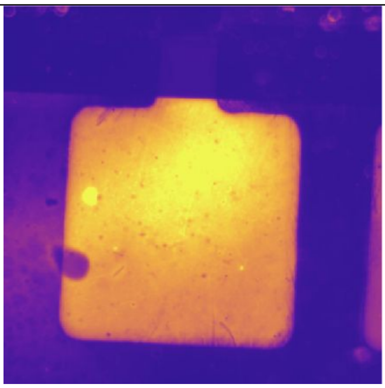
SnO_x device 4<br>Pre-lamination	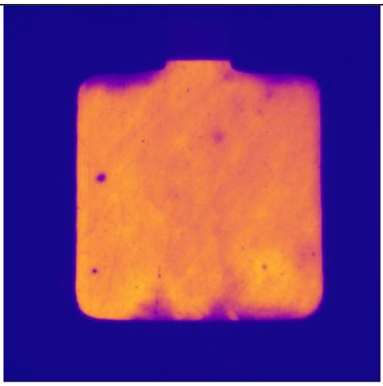	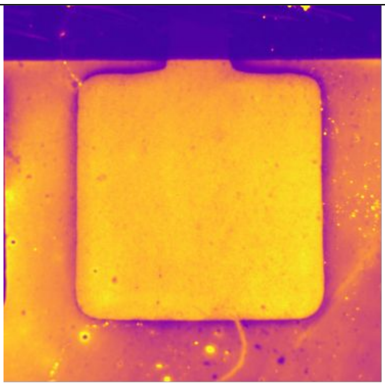
SnO_x device 4<br>Post-lamination	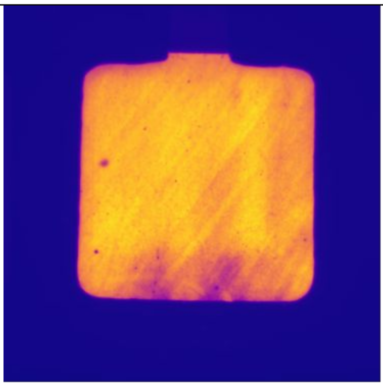	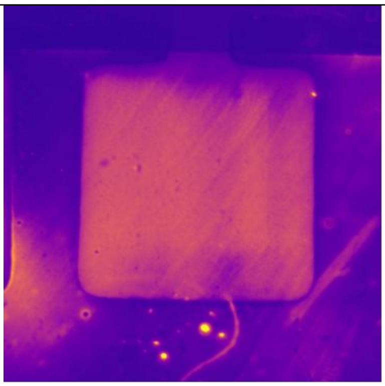

References

(1) Kempe, M. D.; Miller, D. C.; Wohlgemuth, J. H.; Kurtz, S. R.; Moseley, J. M.; Shah, Q. A.; Tamizhmani, G.; Sakurai, K.; Inoue, M.; Doi, T.; Masuda, A.; Samuels, S. L.; Vanderpan, C. E. Field Testing of Thermoplastic Encapsulants in High-Temperature Installations. *Energy sci. eng.* **2015**, *3* (6), 565–580. https://doi.org/10.1002/ese3.104.

(2) Rakotonirina, M. D.; Baron, M.; Siri, D.; Gaudel-Siri, A.; Quinebeche, S.; Flat, J.-J.; Gigmes, D.; Cassagnau, P.; Beyou, E.; Guillaneuf, Y. Acyloxyimide Derivatives as Efficient Promoters of Polyolefin C–H Functionalization: Application in the

Melt Grafting of Maleic Anhydride onto Polyethylene. *Polym. Chem.* **2019**, *10* (31), 4336–4345. https://doi.org/10.1039/C9PY00672A.

(3) Jiao, C.; Wang, Z.; Gui, Z.; Hu, Y. Silane Grafting and Crosslinking of Ethylene–Octene Copolymer. *Eur. Polym. J.* **2005**, *41* (6), 1204–1211. https://doi.org/10.1016/j.eurpolymj.2004.12.008.

(4) Cheacharoen, R.; Rolston, N.; Harwood, D.; Bush, K. A.; Dauskardt, R. H.; McGehee, M. D. Design and Understanding of Encapsulated Perovskite Solar Cells to Withstand Temperature Cycling. *Energy Environ. Sci.* **2018**, *11* (1), 144–150. https://doi.org/10.1039/C7EE02564E.
